# Diverse functions associate with non-coding polymorphisms shared between humans and chimpanzees

**DOI:** 10.1186/s12862-022-02020-x

**Published:** 2022-05-23

**Authors:** Keila Velazquez-Arcelay, Mary Lauren Benton, John A. Capra

**Affiliations:** 1 Department of Biological Sciences, Vanderbilt University, Nashville, TN USA; 2 Department of Computer Science, Baylor University, Waco, TX USA; 3 Departments of Biomedical Informatics and Computer Science, Genetics Institute, and Center for Structural Biology, Vanderbilt University, Nashville, TN USA; 4 Bakar Computational Health Sciences Institute and Department of Epidemiology and Biostatistics, University of California, San Francisco, CA USA

**Keywords:** Trans-species polymorphisms, Balancing selection, Long-term balancing selection, Non-coding variants, Phenome-wide association study

## Abstract

**Background:**

Long-term balancing selection (LTBS) can maintain allelic variation at a locus over millions of years and through speciation events. Variants shared between species in the state of identity-by-descent, hereafter “trans-species polymorphisms”, can result from LTBS, often due to host–pathogen interactions. For instance, the major histocompatibility complex (MHC) locus contains TSPs present across primates. Several hundred candidate LTBS regions have been identified in humans and chimpanzees; however, because many are in non-protein-coding regions of the genome, the functions and potential adaptive roles for most remain unknown.

**Results:**

We integrated diverse genomic annotations to explore the functions of 60 previously identified regions with multiple shared polymorphisms (SPs) between humans and chimpanzees, including 19 with strong evidence of LTBS. We analyzed genome-wide functional assays, expression quantitative trait loci (eQTL), genome-wide association studies (GWAS), and phenome-wide association studies (PheWAS) for all the regions. We identify functional annotations for 59 regions, including 58 with evidence of gene regulatory function from GTEx or functional genomics data and 19 with evidence of trait association from GWAS or PheWAS. As expected, the SPs associate in humans with many immune system phenotypes, including response to pathogens, but we also find associations with a range of other phenotypes, including body size, alcohol intake, cognitive performance, risk-taking behavior, and urate levels.

**Conclusions:**

The diversity of traits associated with non-coding regions with multiple SPs support previous hypotheses that functions beyond the immune system are likely subject to LTBS. Furthermore, several of these trait associations provide support and candidate genetic loci for previous hypothesis about behavioral diversity in human and chimpanzee populations, such as the importance of variation in risk sensitivity.

**Supplementary Information:**

The online version contains supplementary material available at 10.1186/s12862-022-02020-x.

## Significance statement

Most genetic variants present in human populations are young (< 100,000 years old); however, a few hundred are present in both humans and chimpanzees, suggesting that they may be millions of years old with origins before the divergence of these species. Some of these shared polymorphisms were likely influenced by balancing selection—evolutionary pressure to maintain genetic diversity at a locus. In spite of their age, the selected functions, especially for non-coding regions, are largely unknown. We integrate genome-wide annotation strategies to identify candidate non-coding variants likely under long-term balancing selection (LTBS) and find associations with immune system function, behavior (addiction, cognition, risky behavior), uric acid metabolism, and many other phenotypes. These results substantially expand our understanding of functions potentially associated with LTBS and support a role for balancing selection in humans beyond the immune system.

## Background

The interaction between populations and environments is dynamic. Over time, allele frequencies in a population shift due to drift and adaptive responses to specific environmental pressures. Most genetic variants are short-lived compared to the timescale of species. But on rare occasions variants persistently segregate at intermediate frequencies for millions of years, sometimes pre-dating the most recent common ancestor (MRCA) between two sister species [1–6]. These trans-species polymorphisms are often a sign of genomic regions under long-term balancing selection (LTBS). Over time, instances of LTBS leave signatures in the genome that differentiate them from those under other forms of selection [1, 4, 5, 7], such as maintenance of more alleles at intermediate frequency than expected by chance, increased levels of neutral variation near the target site, and deep coalescence times.

Several instances of LTBS regions have been observed in humans and other primates, mostly within the major histocompatibility complex (MHC) or the ABO blood group locus. For example, the MHC, or human leukocyte antigen (HLA) system in humans, is a family of varied proteins expressed on the cell surface with essential functions in adaptive immune response and regulation. Balancing selection on different components of the HLA region dates to the common ancestor between chimpanzees and humans [8–10]. Similarly, the ABO gene has three alleles, and its variants lead to different blood cell antigens, or lack of thereof, on the surface of the cell. Variation in this group could have a benefit in the immune response to pathogens, and balanced polymorphisms at this locus are present in gorillas, orangutans, and humans, and thus likely date back to their last common ancestor [11]. Several other immune-related genes show LTBS between humans and other primates, e.g.: *TRIM5*, a RING finger protein 88 [12–14], and *ZC3HAV1*, a zinc finger CCCH-type antiviral protein 1 [15–18]. These genes have important roles in host/pathogen response through inhibition of virus replication.

The high allelic variation maintained by balancing selection at a locus can also enable adaptation to new environments. For example, some variants found under balancing selection in African and ancestral human populations have experienced directional selection in non-African populations (European and Asian), with one allele becoming predominant in the population [16]. This suggests the adaptive potential of the variation maintained under balancing selection; however, in some cases the adaptive variants themselves may have hitchhiked with those under LTBS.

Recent studies have developed statistical methods to identify instances of balancing selection in genome-wide data [1–3, 5, 19]. Some have focused on detecting LTBS using trans-species data, while others have considered balancing selection over shorter timescales based on single-species data. For example, DeGiorgio [20] developed likelihood-ratio tests (T_1_ and T_2_) based on computing probabilities of polymorphism and substitution under LTBS based on inter-species coalescent modeling to test the spatial distribution of polymorphisms and mutations around genomic sites. With this method they identified balancing selection on HLA regions, but also in a gene that had no previous associations with balancing selection, *FANK1*, which is involved in the suppression of apoptosis during/after the process of meiosis. They also found enrichment for signals in genes with other functions: cell adhesion, membrane protein activity, and components of membranes. A more recent study [2] expanded the T_2_ method to seek trans-species balancing selection without direct consideration of trans-species polymorphism and identified a handful of additional LTBS candidates. Bitarello et al. [1] developed Non-central Deviation (NCD) statistics that quantify the deviation of the local site frequency spectrum (SFS) under balancing selection from neutral expectations. The statistic identifies genomic windows with variants at intermediate frequencies and higher than expected levels of variation as a signature of balancing selection [21]. Applying the statistics to African and European 1000 Genomes populations, they found thousands of candidates for balancing selection in humans. They also showed varying directional selection in different populations, providing evidence for the adaptive potential of regions under balancing selection. Siewert & Voight [5] developed ß, a summary statistic for detecting genomic windows with clusters of intermediate frequency alleles suggestive of balancing selection. They also recently updated the ß statistic to consider both polymorphism and substitution data [19]. Among the highest scoring windows in these two analyses, they highlighted three genes (*CADM2*, *WFS1*, and *ACSBG2*) with functions outside the immune system.

Shared polymorphisms (SPs) between species, especially when more than one falls on a haplotype, suggest the action of LTBS. For example, Leffler et al. [4] compared polymorphisms across the genome in Yoruba individuals from the 1000 Genomes Project to those found in Western chimpanzees sequenced by the PanMap Project. They identified more than 100 non-coding haplotypes with multiple SPs within 4 kilobases (kb) and in high LD as candidates for LTBS. However, sequencing errors and regions with high mutation rates can create patterns that can be mistaken for LTBS. Further modeling has shown that it is unlikely to observe haplotypes with more than two TSPs in close proximity by chance without balancing selection [2, 22].

Despite the importance and prevalence of balancing selection, most of the non-coding haplotypes bearing potential signatures of LTBS (e.g., multiple SPs), have not been functionally characterized. Here, we focus on a high confidence subset of the non-coding SPs identified by Leffler et al. [4]. Determining the candidate functional roles of these SPs in human adaptation and health would deepen our understanding of the dynamics of balancing and positive selection and their roles in adaptation to new environments.

We identify potential functions associated with SP regions in humans by applying several genome-wide functional annotations and association tests. Our results identify diverse functions, including effects unrelated to the immune system, that may have been targets of balancing selection on the human and chimpanzee lineages.

## Results

### Human-chimpanzee shared SNPs

We consider 125 human genomic regions containing multiple variants segregating in both humans and chimpanzees in close proximity and in high LD [4]. The set was defined based on identifying groups of human-chimp shared-polymorphisms (SPs) within 4 kb of each other outside the major histocompatibility (MHC) locus. Based on coalescent theory, this pattern is unlikely to result from neutral processes [4, 11], and these SPs are thus candidates for LTBS (Additional file 1: Fig. S1). However, these criteria alone are insufficient to guarantee that the SPs are the result of identity-by-descent and driven by LTBS [22].

**Fig. 1:**
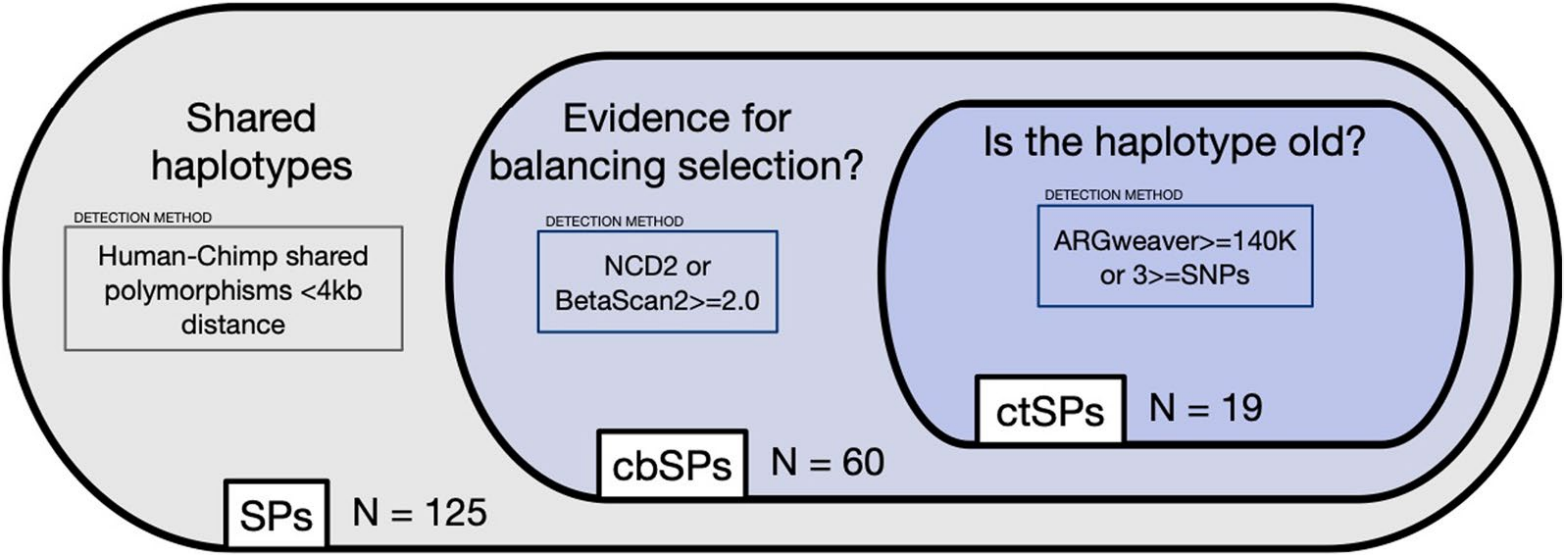
Schematic of the criteria for identifying the SP sets used in this study. A previous study [4] reported a set of 125 candidate regions with two or more non-coding human-chimp shared polymorphisms (SP) within 4 kb. We refined this set based on several additional lines of evidence. First, we considered scores from two balancing selection statistics (NCD and BetaScan2) to create a set of 60 haplotypes with 133 candidate balanced SPs (cbSP). We consider regions with evidence for balancing selection in at least one population from NCD, or regions containing variants with BetaScan2 scores equal or higher than 2.0. We further filtered this set to the 19 haplotypes additionally predicted to be at least 140,000 generations old by ARGweaver or contain at least 3 SPs within 4 kb. These haplotypes include 51 candidate trans-species SP (ctSP) with the highest likelihood of LTBSs

To identify regions with stronger evidence of balancing selection, we consider two additional recent genome-wide balancing selection scans [1, 19] and additional evidence of identity-by-descent (Fig. 1). The first scan is based on NCD, a balancing selection detection statistic that uses the allele frequency spectrum to find regions enriched for intermediate frequency alleles [21]. The second is based on BetaScan2, which detects balancing selection by identifying deviation from neutrality in the vicinity of a haplotype from variance in substitutions and mutation rate. We apply a filter based on regions containing evidence in NCD from at least one population or regions containing at least one SP with a BetaScan2 score of 2.0 or higher. Of the initial set of 125 candidate haplotypes, 60 were highlighted in these recent balancing selection scans. We refer to the 133 variants on these haplotypes as candidate balanced shared polymorphisms (cbSPs). Next, to identify variants with the strongest evidence of LTBS, we further filtered these regions based on additional evidence of human-chimp identity-by-descent to create set of candidate trans-species polymorphisms (ctSPs). For this set, we required the candidate haplotypes additionally to have either extremely ancient times to most recent common ancestor (TMRCA) as estimated by ARGweaver [23] (> 140,000 generations ago) or more than 3 SPs per candidate haplotype. This resulted in 19 haplotypes with 51 ctSPs. In summary, 60 out of the original 125 candidate regions show evidence of balancing selection from at least one of BetaScan2 or NCD (Methods; Additional file 2: Table S1), and 19 of these show additional evidence of identity by descent (Fig. 1).

In the following, we analyze functional annotations and associations for both cbSPs and ctSPs. In some analyses, to capture associations tagged by variants in high linkage disequilibrium (LD) with cbSPs, we also considered potential tag SNPs in high LD (R [2] ≥ 0.8) in African, European, or East Asian populations from the 1000 Genomes Project. This LD-expanded set for cbSPs includes 6,171 variants across the 60 regions (Additional file 1: Figure S2; Additional file 2: Table S2). By expanding to include variants in high LD, we capture additional associations, but may also identify functions unrelated to balancing selection; thus, we report results on both sets.

### Shared polymorphisms overlap diverse functional annotations

**Fig. 2:**
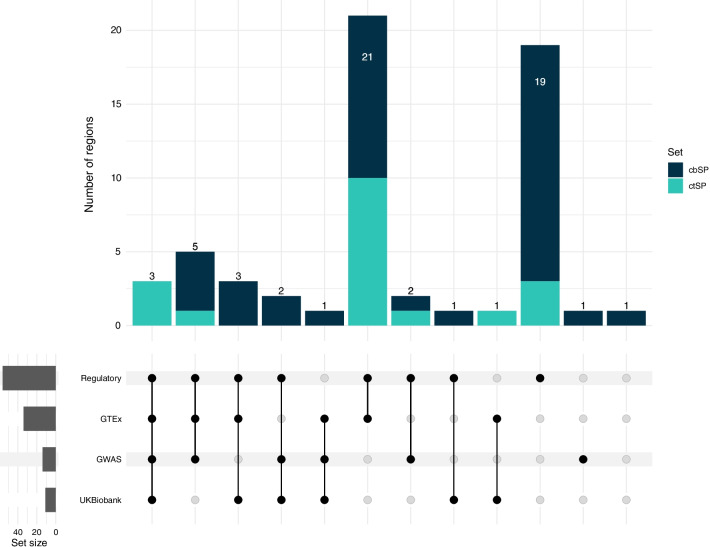
Functional annotations available for the expanded cbSP regions. Summary of the annotations of each type available for cbSP regions, including tagging variants in high LD with cbSPs. A total of 59 out of 60 cbSP regions contain at least one line of functional evidence. The analysis from GWAS, UK Biobank, and regulatory function include annotations for SNPs that are in high LD (0.80 R^2^) with the cbSP set. The UKBiobank set included analysis from geneAtlas and the Neale Lab set. Associations for the highest confidence subset (ctSP regions) are shown in aqua

We intersected the cbSPs with diverse lines of functional evidence from large-scale genomic studies, including genome-wide functional genomics assays, eQTL, GWAS, and PheWAS. We found at least one functional annotation for 98% (59 of 60) of the cbSP regions and all of the ctSP regions, covering 77 SPs and 772 LD SNPs (Fig. 2; Additional file 2: Table S3). Limiting only to the SPs themselves, we found annotations for 68% (41 of 60) of cbSP regions and 84% (16 of 19) of ctSP regions. Here, we provide an overview of the overlap with these annotations. In future sections, we provide details about each of these annotations. Variants in 93% (56 out of 60) of regions overlap annotated gene regulatory regions. This includes 23 cbSPs and 599 LD variants. We also found 64 cbSPs across 34 regions with evidence of being expression quantitative trait loci (eQTL) across 48 tissues. We found genome-wide significant associations with phenotypes in available genome- or phenome-wide association studies in 32% of the LD expanded regions (19 out of 60; 14 GWAS Catalog and 11 UK Biobank from geneAtlas and NealeLab).

### Evidence of gene regulatory function for SPs

We hypothesized that many of the non-coding SPs in our set perform gene regulatory functions. To evaluate this possibility, we intersected the cbSPs and variants in high LD with maps of functional regulatory regions from the Ensembl regulatory build [24]. We found 23 cbSPs with regulatory annotations and additionally 599 LD variants in 56 cbSP regions. These include variants in CTCF binding sites, open chromatin regions, promoter flanking regions, enhancers, promoters, and known TF binding sites (Additional file 2: Table S4). We also tested cbSP regions for enrichment in any specific types of regulatory regions. We compared the observed overlap between cbSP regions and each type of regulatory annotation to the distribution of overlaps expected if cbSP regions were randomly distributed across the genome. We shuffled the cbSP regions 1000 times maintaining their length and chromosome distributions and avoiding genome assembly gaps, ENCODE blacklist regions, and the MHC locus. We compared the number of overlaps observed with regulatory elements with the number from each random permutation (Additional file 1: Figure S3). cbSPs showed more overlap with enhancer and promoter elements than expected, but this was not significant, perhaps due to the small sample size (Additional file 2: Table S5).

**Fig. 3:**
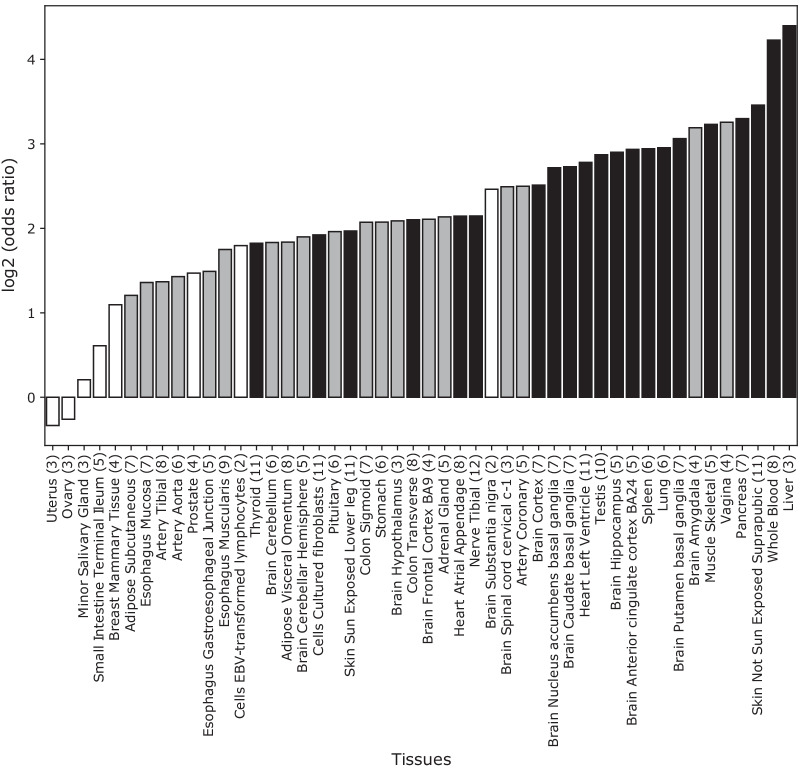
cbSPs are eQTLs in diverse tissues. cbSP regions are enriched for eQTL activity in many tissues compared to genomic background levels (Additional file 2: Table S8). Statistical significance is represented by black, gray, and white bars, where black indicates significance at the Bonferroni correction threshold, gray significance at p < 0.05, and white is not significant. The number of eQTL in the cbSP set for each tissue are given in parentheses following the tissue names. Tissues that are not present had a count of 0 eQTL (i.e. Kidney  cortex)

Overlap of a variant with a regulatory annotation does not necessarily imply a regulatory function. To consider additional evidence of regulatory function, we examined eQTL in GTEx from 50 tissues for overlap with cbSPs. At least one eQTL was found for 34 of the regions (57%). Among these 34 regions, 64 cbSPs are themselves eQTL in 48 tissues (Additional file 2: Table S6). We tested for enrichment of eQTL in cbSPs compared to the background across all genomic regions and found enrichment for eQTL activity in a diversity of GTEx tissues, including liver, whole blood, skin, and pancreas (Fig. 3).

We found diverse gene ontology (GO) terms among the genes influenced by cbSP eQTL, but no individual terms remained significant after multiple testing correction (Additional file 2: Table S7). These results suggest that the targets of balancing selection in these regions may have functions in gene regulation across diverse tissues beyond the immune system (Additional file 2: Table S8).

### Genome-wide association studies link cbSPs to traits

**Fig. 4:**
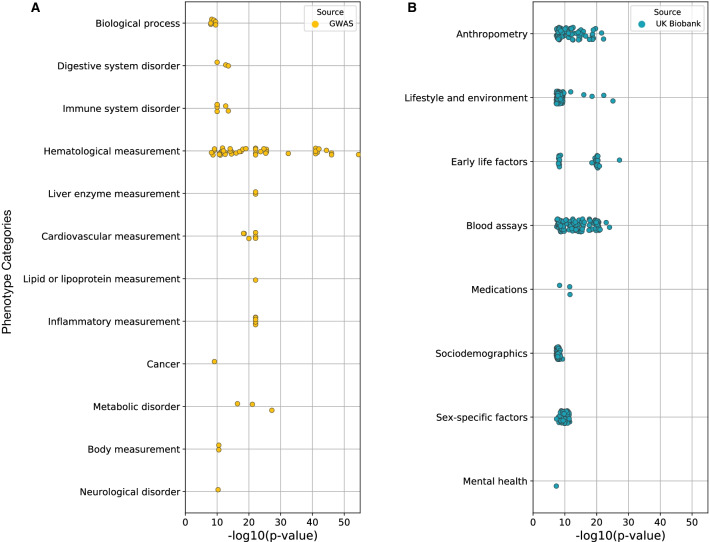
Genome- and phenome-wide association studies link cbSPs to diverse traits. Genome-wide significant (P < 1E−8) associations from the GWAS Catalog (**A**) and PheWAS over the UK Biobank (**B**) from the geneAtlas and NealeLab [26]. Each dot represents an association between a cbSP region and a trait. Many immune-related traits (under immune system disorder, blood assays, and other measurements) are associated with cbSPs, but there are also associations with a wider variety of phenotypes including lifestyle and environment, neurological traits, and cognitive performance. Since few cbSPs themselves were directly tested in GWAS, we include GWAS Catalog associations with tag variants in high LD (r^2^ > 0.8) with the cbSPs. We also observed associations in the "other measurements" and “other disease” parent categories, which include miscellaneous measurements and traits that did not fit in the listed categories. All associations are given in Additional file 2: Tables S9 and S10. For the most enriched GWAS categories, see Additional file 1: Figure S4 and Additional file 2: Table S11

Genome-wide association studies have identified thousands of associations between genetic variants and human traits. We intersected the cbSP regions with associations reported in the GWAS Catalog (downloaded 2021/12), which is composed of over 170,000 associations in 4,070 terms. Since cbSPs themselves were not always directly tested in GWAS, we also include genome-wide significant (p <  = 5E−8) associations with the tag variants in high LD with SPs. We found significant associations for 52 different variants (Fig. 4A; Additional file 2: Table S9). Among the functional associations we found immunological functions, hematological/blood measurements, and anthropometric traits. The associations with immune traits were expected given the results of previous balancing selection studies and the few well-characterized instances of LTBS. We identified many variants in LD with cbSPs that are associated with blood measurement phenotypes and diseases related to immune response (Additional file 2: Tables S3 and S9). These traits include ulcerative colitis and other chronic inflammatory diseases (chr2 near cbSPs rs13426764/rs11694806).

We also found many neurological and behavior-related associations among cbSP region variants. These traits include cognitive performance (rs13426764 and rs11694806 on chromosome 2 and rs9869178/rs2118072 on chromosome 3), alcohol and smoking status (alcohol use: chromosome 16 near rs9933768 and rs57790054; smoking: chromosome 2 near rs13426764 and rs11694806), risky behavior (automobile speeding propensity: chromosome 3, rs9869178/rs2118072), experiencing mood swings (chromosome 2 near rs13426764 and rs11694806), insomnia, neuroticism, sun-seeking behavior, and age at first sexual intercourse (Additional file 2: Table S9). In addition to the immune response and neurological categories, we observed associations in reproductive traits (polycystic ovary syndrome, testosterone levels), urate levels, pancreatic cancer, and gut microbiota. An enrichment analysis found significant results for GWAS categories including blood and immune related traits, uric acid levels (including urate and gout), cognitive performance measurements (intelligence, educational attainment, math ability), smoking status, and gut microbiome measurement (Additional file 1: Fig. S4). We discuss several of these associations in more detail in following sections.

### Phenome-wide association studies link cbSPs to additional diverse traits

The growth of biobanks with linked genetic and phenotypic data has enabled the testing of the association of genetic variants with diverse traits within a single cohort. This PheWAS approach enables exploration of the functional and potentially pleiotropic effects of variants of interest [25]. Using published associations from the UK Biobank (geneAtlas and NealeLab), we analyzed the association of cbSPs with over a thousand traits; all 60 of the regions were tested. Overall, we found that 150 different variants in 11 regions had at least one genome-wide significant association (P < 1E–8, Fig. 4B; Table S10). Though testing different phenotypes than the GWAS, these associations were qualitatively similar to the GWAS results, in that blood and immune system phenotypes had many associations with cbSPs, but the cbSPs were also associated with a more diverse set of phenotypes. We found associations in many categories including blood assays, body measurements, and lifestyle/environment traits. Among the observed associations we found, for example: hair color, standing height, number of days/week walked 10 + minutes, and 28 variants associated with alcohol intake frequency (Additional file 2: Tables S3 and S10).

### Illustrative examples of diverse functions associated with cbSP regions

Integrating the above data, we found 38 cbSP regions with two or more lines of functional evidence (Fig. 2). This includes 13 regions with annotations from at least three evidence sources. To illustrate the diverse functions associated with cbSPs, we highlight three of these regions (Additional file 2: Table S1) [1, 19]. In these detailed analyses, we also considered additional manually identified annotations and associations from the literature and sources like the gwasAtlas [26].

**Fig. 5:**
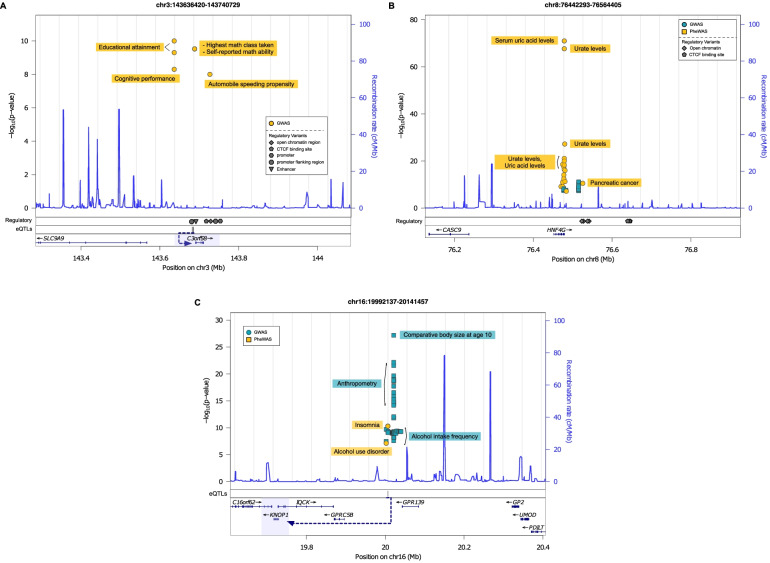
Illustrative examples of non-immune functions associated with cbSPs. **A** LD SNPs in ctSP locus on 3q24 is associated with cognitive performance and risky behavior. Regional association plot showing statistically significant genome- and phenome-wide associations (threshold p ≤ 1E−08), regulatory and eQTLs. This locus is characterized by neurological traits involved in educational attainment, cognitive performance, and risky behavior (automobile speeding propensity). Both ctSPs in this region (rs9869178, rs2118072) are eQTL in the gene *DIPK2A* (*C3orf58*). LD SNPs are found in enhancer and promoter flanking regions. **B** SNPs in high LD with cbSPs in 8q21.11 are associated with uric acid and urate levels. Regional association plot showing statistically significant genome- and phenome-wide associations (P ≤ 1E−08), eQTL, and regulatory (open chromatin, CTCF binding site) SNPs. LD SNPs in this region are associated with urate (rs2941484, rs2943539) and uric acid (rs2977944, rs2941484) levels, and pancreatic cancer (rs2941471, p = 7E−10). **C** A cbSP in 16p12.3 is associated with alcohol intake frequency and comparative body size at age 10. The regional association plot shows statistically significant genome- and phenome-wide associations (threshold p ≤ 1E−08), and eQTLs from GTEx. One of the cbSPs (rs57790054, yellow) is associated with alcohol intake in the UK Biobank. A variant in high LD (rs72771074, green) has been associated with alcohol use disorder in a previous GWAS. The cbSP is also strongly associated with insomnia (5e-11). The cbSPs are nearby *GPR139*, a gene encoding a G-protein coupled receptor expressed in the brain, whose expression levels influence alcohol drinking behavior in rats. Figures created with LocusZoom [40]

*Risky behavior and cognitive performance*. A ctSP region on chromosome 3q24 is more than 235,000 generations old, and thus has strong evidence of identity by descent between humans and chimpanzees. Both ctSPs in this region (rs9869178, rs2118072) are associated with a risky behavior: automobile speeding propensity (Additional file 2: Table S12). The ctSPs are also modestly associated with variation in brain white matter microstructure (Anterior corona radiata mean diusivities, P = 1.96E–6) [27], as reported in the gwasAtlas database. Variants in the expanded ctSPs region in 3q24 (hg19.chr3:143636420–143740729) are associated with risky behavior and cognitive performance traits in multiple individual GWAS (Fig. 5A). For example, they are associated with automobile speeding propensity (P = 1E−8) [28], cognitive performance (P = 5E−9), educational attainment (P = 1E–10) [29], and self-reported math ability and highest math class taken (both P = 3E–10). Many of the variants in high LD with the ctSPs in this region overlap annotated regulatory regions: open chromatin region, promoter, promoter flanking region, CTCF binding sites, and enhancer (Additional file 2: Table S4). Furthermore, the ctSPs are significant eQTLs (P ≤ 1E−5) for the gene *DIPK2A* (*C3orf58*) across four GTEx tissues (small intestine terminal ileum, transformed fibroblasts, skin from the lower leg, and suprapubic skin). The DIPK2A protein has not been comprehensively functionally characterized, but it contains a protein kinase domain and is broadly expressed, including in the developing and adult brain. Deletion of this gene has been linked to autism, and its expression is responsive to neuronal activity [30].

*Urate levels.* Two cbSPs (rs1839333, rs1913638) on chromosome 8q21.11 are both significantly associated (P < 2.0e−18, Additional file 2: Table S12) with uric acid levels in multiple GWAS in European and Asian ancestry populations (Fig. 5B) [31–33]. These variants are also associated with a range of body mass traits in the UK Biobank. Another variant in this locus (rs2941471, R^2^ = 0.97 and R^2^ = 0.82 in East Asians and Europeans respectively) is associated with pancreatic cancer (p = 7E−10). Though elevated uric acid in the blood is associated with many conditions, it is a marker for pancreatic cancer [34]. This locus also contains LD SNPs (rs1805098 and rs2943549) in East Asians that are expression and splicing QTL for the gene *HNF4G* in testis, pancreas, and brain (P ≤ 5E−5). Variants in *HNF4G* are associated with several traits, including the development of hyperuricemia [35]. One of the cbSPs (rs1839333, p = 2.65E−05) is also associated with gout, although the p-value did not meet our strict threshold.

*Body mass and alcohol intake*. A cbSP (rs57790054) on 16p12.3 (hg19.chr16: 20006097–20006986) is strongly associated with several growth and body mass phenotypes as well as alcohol intake frequency (Fig. 5C; P < 5E−8 for all). Another variant in high LD in Europeans (rs72771074, R^2^ = 0.89) with a cbSP (rs57790054) in this locus was associated with alcohol use disorder in a previous GWAS in a European cohort (P = 5E−8) [36]. The nearest gene, *GPR139*, encodes for a G-protein coupled receptor expressed in the brain that is involved in alcohol drinking behavior and withdrawal symptoms in rats [37]. This region contains several variants in LD with cbSPs in regulatory regions, such as CTCF binding sites (rs117293173, rs13338055, rs74011247, and rs79521770). One cbSP (rs57790054, p = 1.89E−5) is an eQTL for the gene *KNOP1* (aka *C16orf88*). This gene has been associated with obsessive compulsive disorder, among other diseases [38]. These results suggest that effects on growth and BMI or on addictive behaviors could be under LTBS. We note that there is some evidence of ethanol consumption in chimpanzees, but it is unclear how widespread its availability was over the past several million years [39].

## Discussion

In this study we aimed to characterize the function of genomic regions with multiple lines of evidence of LTBS on the human lineage. We started with candidate regions containing two or more human-chimp SPs in LD and close proximity. We then considered additional evidence from genome-wide scans for balancing selection with BetaScan2 and NCD, and allele age estimates from ARGweaver. Variants in the resulting candidate sets likely have deep ancestry in the common ancestor between humans and chimpanzees and have persisted in the genomes of both species for millions of years. However, the majority of the non-coding candidate LTBS regions previously identified do not have known functions.

We addressed this challenge with the help of newly developed genomic annotation tools and identified at least one functional annotation for 59 out of 60 cbSP regions and all the ctSP regions. These annotations suggest that non-coding SPs likely maintained by LTBS have diverse functions beyond enabling a flexible immune response to pathogens. This expands on several recent studies of balancing selection over shorter timescales that have also identified regions with functions outside the immune system [1, 5, 41, 42].

To explore the gene regulatory potential of cbSPs, we analyzed eQTL data from 48 tissues from the GTEx Atlas. We found that cbSPs are often eQTL for genes in tissues beyond the immune system, and we observed significant enrichment for eQTL activity in diverse tissues, including many brain and reproductive tissues. A recent study of genes potentially evolving under LTBS identified by the NCD2 statistic found enrichment for genes expressed in the lung, adipose tissue, adrenal tissue, kidney, and prostate [1]. Among our non-coding candidate regions, there is significant enrichment in lung, nominally significant enrichment for adipose and adrenal tissues, and none for prostate or kidney (Fig. 3). These differences suggest that the functions of coding vs. non-coding regions subject to LTBS may differ. However, we note that the number of regions considered in each analysis is relatively small.

The phenotype associations we observe for candidate variants in GWAS and PheWAS suggest possible behavioral, neurological, and morphological traits that may be targets of LTBS. In particular, our results provide support and candidate loci for previous hypotheses about the need for neurological and behavioral diversity in populations. For example, we found evidence for association with risky behavior and cognitive performance in one ctSP region. Selection has recently been shown to act on risk-taking behavior in anole lizards [43]. Thus, our identification of associations between ctSPs and human risk-taking behavior (Fig. 4A) suggests that LTBS may have maintained genetic variants that contribute to variation in risk taking behavior in humans and chimpanzees. The ctSPs are eQTL for *DIPK2A* (*C3orf58*), which encodes for a protein kinase and has been associated with autism and other neurological disorders [44]. Associations with behavioral and cognitive traits must be interpreted with caution as these traits are very challenging to quantify and strongly influenced by social factors that may vary with other characteristics. Nonetheless, these associations point to an influence of the ctSPs on behaviors relevant to risk tolerance. Thus, it is possible that maintaining a diversity of risk tolerance in human and chimpanzee populations has been beneficial.

Our results also raise the intriguing possibility that variants that modulate urate levels have been under LTBS. Uricase, the enzyme that metabolizes uric acid into an easily excreted water-soluble form in most mammals, has been lost in great apes. This gene was disabled by a series of mutations that slowly decreased activity over primate evolution, increasing the levels of uric acid in blood [45, 46]. It has been hypothesized that this loss of uricase activity was driven by increase fructose in primate diets due to fruit eating [45, 47]. It has also been proposed that high levels of uric acid, a potent antioxidant, played an important role in the evolution of intelligence, acting as an antioxidant in the brain [48]. However, as reflected in the associations with this locus, elevated uric acid levels contribute to many common diseases in modern humans, including chronic hypertension, cardiovascular disease, kidney and liver diseases, metabolic syndrome, diabetes, and obesity [49]. This suggests potential functional tradeoffs at this locus; however, proving the environmental drivers of past selection is challenging.

Some of the phenotype associations we discovered may reflect manifestations of variation on traits in modern environments that could not be long-term drivers of balancing selection. As an extreme example, influence on smoking behavior could not have been the cause of LTBS given the relatively recent wide availability of nicotine. Though we note that there is some evidence of ethanol consumption in chimpanzees [39]. Even if they reflect modern environments, these associations provide hints about possible behavioral, neurological, or other traits that may have driven LTBS. For instance, plant chemicals can hijack reward systems in the brain that motivate repetition and learning [50]. The same systems that influence these actions and consequently reproductive fitness could potentially be a byproduct of excessive seeking of dopamine or other reward chemicals.

There are several caveats to our work. First, factors other than LTBS, such as high mutation rates and sequencing errors, can produce signals similar to those of LTBS. However, our use of additional evidence from balancing selection detection methods, and filters by evidence of ancient origins or the presence of multiple cbSPs in the regions we considered strongly suggest LTBS. Nonetheless, candidate regions of interest for future study should be further analyzed for possible confounders. Moreover, additional approaches for identifying signatures of LTBS have recently been developed. For example, the T_2,trans_ statistic has been shown to have higher power than single species metrics in many scenarios [2]. Considering this metric in the definition of cbSPs only identified one additional locus (defined by rs16872492, rs114975228), and it did not have clear functional annotations. Future work will likely identify additional candidate regions that could be characterized using our approaches.

Even with recent growth of genetic and phenotypic databases, our knowledge of the functions of most regions of the genome is sparse. Thus, failure to observe a functional association does not imply that a region does not have an important function. The genome- and phenome-wide association tools we used are limited to the samples that have been analyzed; available data do not represent the full scope of human variation. Most of the individuals analyzed in available genetic association studies are of European ancestry [51]. Variant functions and the ability to detect associations vary across human populations; however, we anticipate that SPs should have functional effects across populations, unless modern environments have masked the pressure driving LTBS. Even in PheWAS, a limited number of phenotypes have been quantified across individuals, and these studies are focused on a subset of clinically relevant rather than evolutionarily relevant traits. To expand the potential to identify candidate functions, in some analyses we considered annotations based on trait associations with variants in high LD (r^2^ > 0.8) with cbSPs. This could potentially introduce false positives if the variant also tags a different causal variant that is not subject to LTBS. However, these associations would still implicate the regions with signatures of LTBS in the associated functions, but functional studies are needed to confirm the role of the candidate variants in these associations. Finally, our analyses have focused on the human context. Due to lack of functional data, it is not possible to explore the function of cbSPs in chimpanzees. Nonetheless, we feel that our integration of genome-scale annotations and biobank data highlights the diversity of functions associated with LTBS.

## Conclusions

In conclusion, we assign putative functions to many non-coding haplotypes carrying human-chimpanzee SPs that likely persisted due to balancing selection dating back to at least their common ancestor. These annotations expand beyond immune functions to traits relevant to behavior, cognition, and body shape. Notably, we also find that most regions with multiple cbSPs overlap gene regulatory annotations suggesting balancing selection on gene expression levels. As methods improve for quantifying the effects of variants on gene regulation in different tissues and how these relate to organism-level phenotypes, we anticipate deeper mechanistic understanding of the functions and potential evolutionary pressures on these regions.

## Methods

### Human-chimpanzee shared polymorphisms and balancing selection scans

The initial set of 125 regions containing 263 human-chimp shared polymorphisms analyzed in this study was published by Leffler et al. [4]. The set is composed of regions that: (1) contain at least two trans-species polymorphisms—i.e., variants that are segregating in both 51 Yoruba individuals in the 1000 Genomes Pilot 1 and 10 chimpanzees from the PanMap project—within 4 kb of each other in both species, and (2) are in high LD in humans and chimpanzees.

We overlapped the shared polymorphism (SP) regions with balancing selection candidate regions from two different methods developed to detect balancing selection. BetaScan2 [19] is a statistic for detecting balancing selection based enrichment for variants in a region with low variation in allele frequency and a deficit of substitutions. We identified overlaps between the SP regions and genomic regions detected by BetaScan2. Among the regions with Beta scores, 48% (60/125) had a SP with value greater than the 2.0 standardized beta score threshold used by the authors. We also computed overlap with regions identified by the NCD statistic [1]. The overlap with the regions detected by NCD containing evidence from at least one population is 14% (18/125 regions). In total, 48% (60/125) of the SP regions were supported by either the BetaScan2 or NCD. We refer to the resulting set of 60 regions as candidate balanced shared polymorphism (cbSP) regions.

### Candidate trans-species polymorphisms

We further filtered the cbSP set to find high-confidence candidate trans-species balanced shared polymorphisms (ctSPs). To achieve this, we first selected all cbSP regions that contain three or more SPs, since this is estimated to substantially reduce the false positive rate [22]. We additionally considered time to more recent common ancestor (TMRCA) predictions for the cbSPs from an ancestral recombination graph method, ARGweaver [23]. ARGweaver reconstructs the recombination history of a genomic site and estimates its age. Following the threshold used in the original ARGweaver analysis of LTBS candidate regions, we filtered cbSP regions to those that are estimated to be 140,000 generations or older, and thus approach the human-chimpanzee divergence. The ctSP subset contains 19 cbSPs.

To increase our ability to identify trait annotations in each locus, we also created an expanded set that includes variants in high LD (threshold R^2^ = 0.8) with each of the SPs as is common in association studies. We computed linkage disequilibrium for the SP variants from 1000 Genomes Project Phase 3 data using the SNiPA Proxy Search web tool developed by the German Research Center for Environmental Health (https://snipa.helmholtz-muenchen.de/snipa3/). We considered LD in African, East Asia, and European populations. Variants with no reported RSID name were excluded from the analysis. The dataset was thus expanded by 6,038 SNPs in high LD with the cbSPs for a total of 6,171 SNPs.

### Genome- and phenome-wide associations

The GWAS Catalog (https://www.ebi.ac.uk/gwas/) collects variant-trait associations from published genome-wide association studies. The database is currently composed of more than 200,000 associations. We used the GWAS Catalog (download date: December 2021) to find functional associations for the LTBS variants. The search was done using the BEDTools intersect function between the GWAS catalog and the LD-expanded SP dataset [52].

We performed an enrichment analysis for Experimental Factor Ontology (EFO) trait categories associated with cbSPs in the GWAS catalog using a binomial test based on the background probability of each category across the full catalog. We apply a Bonferroni correction for the number of EFO terms tested (0.05/394 categories tested). However, given the small number of associations with any specific trait, relative enrichment is challenging to quantify.

PheWAS is an analysis strategy built on top of medical records with information about patient phenotypes and associated variants. The geneAtlas (http://geneatlas.roslin.ed.ac.uk/) and the NealeLab (http://www.nealelab.is/uk-biobank) catalogs take advantage of the data provided by the UK Biobank cohort, which contains medically relevant data from nearly 500,000 British individuals of European ancestry. The geneAtlas database contains 3 million variants in 778 traits and the NealeLab database contains more 50,000 variants in more than 4000 phenotypes. We matched our set of variants against these databases to search for traits associated with balancing selection.

### GTEx eQTL data

To evaluate potential gene regulatory effects of SPs in non-coding regions, we analyzed data from GTEx, a project developed to quantify the consequence of genetic variation on expression at the tissue level (https://www.gtexportal.org/). The GTEx project v8 data have identified eQTL across 50 tissues based on analyses of nearly 1000 individuals to identify differential expression through SNP variation. The intersection between the SPs and LD SNPs and the GTEx eQTL returned a large collection of SPs with evidence of eQTL. To explore the patterns of the cbSPs on regulatory function, we performed an enrichment analysis on these results by calculating the odds ratio on the number of eQTLs for each tissue in the GTEx catalog.

### Enrichment for overlap with regulatory regions

We used a permutation framework to calculate whether SPs were more enriched for overlap with regulatory regions than expected by chance [53]. We quantified the number of overlapping SPs for each type of regulatory region (open chromatin, promoter, enhancer, promoter-flanking, CTCF binding site, TF binding site). We then compared the observed SP overlap to a null distribution of expected overlap generated by randomly shuffling the regulatory regions 1000 times across the genome. We maintain the original length and chromosome distributions for shuffled regions and exclude all ENCODE blacklist and gap regions [54], as well as the human MHC locus, since SPs in this region were excluded from the Leffler et al. set. We then computed an empirical p-value for the observed SP overlap based on the distribution of overlaps for the set of matched shuffled regions.

## Supplementary Information

**Additional file 1: Figure S1. **Human-chimpanzee shared polymorphisms (SPs) previously reported as candidate targets of long-term balancing selection (LTBS). Schematic showing the criteria used by Leffler et al. (2013) to identify SPs likely maintained by LTBS. Each line represents a chromosome with polymorphisms segregating in a species. A/A’ are two alleles segregating in both humans and chimpanzees at one site (i.e., an SP), and B/B’ are two alleles segregating in both species at a nearby SP site. SPs are very unlikely to appear nearby (within 4 kb) without the action of balancing selection. Within these regions, multiple functional scenarios are possible. For example, one SP may be under LTBS while the other is neutral, but maintained due to tight linkage. Alternatively, the SPs may have epistatic functions and both be under selection.** Figure S2.** SNPs in LD with candidate balanced shared polymorphisms (cbSPs). We consider 60 regions containing 133 cbSPs. For each of these SNPs we find variants in high LD (R^2^ >= 0.8). As a result, we obtain an additional 6,038 LD variants from the 1000 Genomes Project. Counts include LD SNPs and cbSPs. Figure created with www.biovenn.nl. **Figure S3.** Enrichment analysis of cbSP in annotated regulatory regions. cbSPs overlap more enhancers promoters, and open chromatin regions and fewer CTCF binding sites than expected compared to length- and chromosome-matched non-coding regions from the genomic background. However, these signals were not statistically significant. Enrichment was tested in the cbSP haplotype region (A) and in the LD region (B). Since variants in CTCF regions are likely to influence regulation of many genes in many tissues (e.g., compared to enhancers which are often context-specific), this suggests that individual cbSPs may be less pleiotropic than expected by chance. C) The proportion of LD variants observed in each regulatory feature type (bottom) and genome-wide (top). **Figure S4.** Enrichment analysis of GWAS phenotype categories. (Top) We performed an enrichment analysis on the GWAS phenotype categories (EFOs) and found significant enrichment in many of the categories. Bars colored in gray meet a significant threshold of 0.05 P-value (binomial test), and bars colored in black pass a Bonferroni correction. (Bottom) The most enriched GWAS EFO categories include blood and immune related traits, and also cognitive, smoking status, and uric acid related traits, including urate levels and gout. All the categories represent a significant enrichment under a Bonferroni correction (binomial test). However, we note that the absolute number of associations driving these enrichments are very small.

**Additional file 2: Table S1.** List of candidate balanced shared polymorphisms (cbSPs), including subset of candidate trans-species shared polymorphisms(ctSP). **Table S2.** cbSPs and SNPs in high LD (R2 >= 0.8). **Table S3.** Summary table of the variants in this study, statistics, and associations. **Table S4.** Regulatory (VEP) association results for set of cbSPs and SNPs in high LD (R2 >= 0.8). **Table S5.** Regulatory region permutation test. **Table S6.** GTEx association results for set of cbSPs. The P-Value threshold reported here is 5e-5. **Table S7.** Gene Ontology (WebGestalt) performed on GTEx genes that contain cbSP eQTLs (Table S6) did not return any significan terms. **Table S8.** GTEx background probability of cbSPs. Bonferroni correction values represent: 2-passed the test, 1-p-value above 0.05, 0-not significant. **Table S9.** GWAS associations for cbSP and LD variants. The P-Value threshold reported here is 5e-8. **Table S10.** UKBiobank (geneAtlas and NealeLab) associations for cbSP and LD variants. The P-Value threshold reported here is 5e-8. **Table S11.** GWAS background probability of cbSPs. Bonferroni values represent: 2-passed the test, 1-p-value above 0.05, 0-not significant. **Table S12.** cbSPs found in association studies from gwasAtlas database. The P-Value threshold reported here is 5e-8.

## Data Availability

The data underlying this article are available in the article and in its online Additional files.

## Abbreviations

LTBS: Long-term balancing selection
TSP: Trans-species polymorphisms
SP: Shared polymorphisms
ctSP: Candidate trans-species polymorphisms
cbSP: Candidate balanced shared polymorphisms
LD: Linkage disequilibrium
eQTL: Expression quantitative trait loci
GTEx: Genotype-tissue expression
GWAS: Genome-wide association study
PheWAS: Phenome-wide association study
Shared polymorphisms (SPs): Leffler SNPs in the 125 regions
Candidate balancing selection SPs (cbSPs): SNPs in 60 Leffler regions with BS evidence
Candidate trans-species shared polymorphisms (ctSPs): SNPs in the 19 Leffler regions with BS evidence and 3 + Leffler SNPs and/or ARGweaver old
