# Supplementary material for: Diverse functions associate with non-coding polymorphisms shared between humans and chimpanzees
Source: BMC Ecol Evol. 2022 May 23;22:68. doi: 10.1186/s12862-022-02020-x (PMC9125839; doi:10.1186/s12862-022-02020-x)
Supplement: Supplementary file 1 — Additional file 1: Figure S1. Human-chimpanzee shared polymorphisms (SPs) previously reported as candidate targets of long-term balancing selection (LTBS). Schematic showing the criteria used by Leffler et al. (2013) to identify SPs likely maintained by LTBS. Each line represents a chromosome with polymorphisms segregating in a species. A/A’ are two alleles segregating in both humans and chimpanzees at one site (i.e., an SP), and B/B’ are two alleles segregating in both species at a nearby SP site. SPs are very unlikely to appear nearby (within 4 kb) without the action of balancing selection. Within these regions, multiple functional scenarios are possible. For example, one SP may be under LTBS while the other is neutral, but maintained due to tight linkage. Alternatively, the SPs may have epistatic functions and both be under selection. Figure S2. SNPs in LD with candidate balanced shared polymorphisms (cbSPs). We consider 60 regions containing 133 cbSPs. For each of these SNPs we find variants in high LD (R2 >= 0.8). As a result, we obtain an additional 6,038 LD variants from the 1000 Genomes Project. Counts include LD SNPs and cbSPs. Figure created with www.biovenn.nl. Figure S3. Enrichment analysis of cbSP in annotated regulatory regions. cbSPs overlap more enhancers promoters, and open chromatin regions and fewer CTCF binding sites than expected compared to length- and chromosome-matched non-coding regions from the genomic background. However, these signals were not statistically significant. Enrichment was tested in the cbSP haplotype region (A) and in the LD region (B). Since variants in CTCF regions are likely to influence regulation of many genes in many tissues (e.g., compared to enhancers which are often context-specific), this suggests that individual cbSPs may be less pleiotropic than expected by chance. C) The proportion of LD variants observed in each regulatory feature type (bottom) and genome-wide (top). Figure S4. Enrichment analysis of GWAS phenot [file 12862_2022_2020_MOESM1_ESM.docx]

**Additional Figures**

**Figure S1. Human-chimpanzee shared polymorphisms (SPs) previously reported as candidate targets of long-term balancing selection (LTBS).** Schematic showing the criteria used by Leffler et al. (2013) to identify SPs likely maintained by LTBS. Each line represents a chromosome with polymorphisms segregating in a species. A/A’ are two alleles segregating in both humans and chimpanzees at one site (i.e., an SP), and B/B’ are two alleles segregating in both species at a nearby SP site. SPs are very unlikely to appear nearby (within 4 kb) without the action of balancing selection. Within these regions, multiple functional scenarios are possible. For example, one SP may be under LTBS while the other is neutral, but maintained due to tight linkage. Alternatively, the SPs may have epistatic functions and both be under selection.


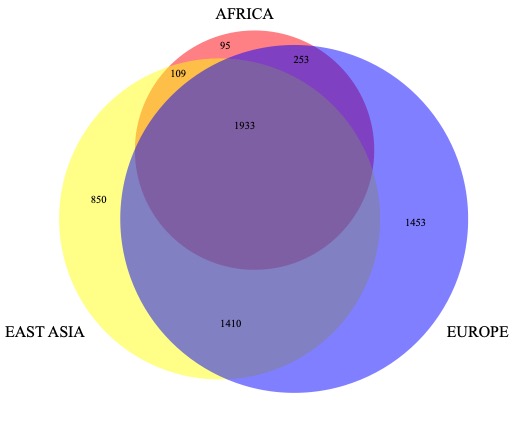


**Figure S2. SNPs in LD with candidate balanced shared polymorphisms (cbSPs).** We consider 60 regions containing 133 cbSPs. For each of these SNPs we find variants in high LD (R^2^ >= 0.8). As a result, we obtain an additional 6,038 LD variants from the 1000 Genomes Project. Counts include LD SNPs and cbSPs. Figure created with www.biovenn.nl

**Figure S3: Enrichment analysis of cbSP in annotated regulatory regions.**

cbSPs overlap more enhancers promoters, and open chromatin regions and fewer CTCF binding sites than expected compared to length- and chromosome-matched non-coding regions from the genomic background. However, these signals were not statistically significant. Enrichment was tested in the cbSP haplotype region (A) and in the LD region (B). Since variants in CTCF regions are likely to influence regulation of many genes in many tissues (e.g., compared to enhancers which are often context-specific), this suggests that individual cbSPs may be less pleiotropic than expected by chance. C) The proportion of LD variants observed in each regulatory feature type (bottom) and genome-wide (top).

**Figure S4: Enrichment analysis of GWAS phenotype categories.** (Top) We performed an enrichment analysis on the GWAS phenotype categories (EFOs) and found significant enrichment in many of the categories. Bars colored in gray meet a significant threshold of 0.05 P-value (binomial test), and bars colored in black pass a Bonferroni correction. (Bottom) The most enriched GWAS EFO categories include blood and immune related traits, and also cognitive, smoking status, and uric acid related traits, including urate levels and gout. All the categories represent a significant enrichment under a Bonferroni correction (binomial test). However, we note that the absolute number of associations driving these enrichments are very small.
